# Comparison of three different anastomotic methods of sleeve gastrectomy with transit bipartition using an obese rodent model

**DOI:** 10.1038/s41598-023-48059-8

**Published:** 2023-12-01

**Authors:** Yuxiao Chu, Jason Widjaja, Jian Wang, Wei Wu, Jian Hong, Xiaocheng Zhu, Libin Yao

**Affiliations:** 1 Department of Gastrointestinal Surgery, The Affiliated Hospital of Xuzhou Medical University, Xuzhou, 221002 Jiangsu People’s Republic of China; 2 Department of General Surgery, Fudan University Affiliated Huadong Hospital, Shanghai, 200040 People’s Republic of China

**Keywords:** Diseases, Medical research

## Abstract

The long-term effects and safety of single-anastomosis sleeve ileal (SASI) bypass have not been confirmed. The one anastomosis procedure carries the risk of bile reflux, and Braun anastomosis has the capacity to reduce bile reflux. This study was designed to compare the influences of bile reflux and histological changes in the esogastric sections of rats. Obese Sprague–Dawley rats underwent sleeve gastrectomy with transit bipartition (RYTB) (n = 12), SASI (n = 12), SASI bypass with Braun anastomosis (BTB) (n = 12), esojejunostomy (EJ) (n = 12), and SHAM (n = 8) surgery. During the 12-week follow-up period, weight changes, glucose improvement, and changes in serum nutrition were evaluated. Histological expression and bile acid concentration in the rats in all groups were also evaluated. No significant differences in weight loss and glucose improvements were observed in the RYTB, SASI, and BTB groups. The RYTB and BTB groups had significantly lower bile acid concentration and albumin levels than the SASI group. In addition, mucosal height in the RYTB and BTB groups was significantly lower than in the SASI group. Braun anastomosis had a significant effect on anti-reflux. BTB may be a superior primary procedure due to its potential for parallel bariatric and metabolic improvements, effective anti-reflux effects, simplified operations, and avoidance of severe malnutrition. Further clinical studies are needed to confirm these findings.

## Introduction

Type 2 diabetes mellitus (T2DM) and severe obesity have become public health issues worldwide^1^. The most efficient treatment for severe obesity complicated by T2DM is bariatric surgery^2^.

Sleeve gastrectomy with transit bipartition (SG-TB), proposed in 2012^3^, is a novel procedure that has been proven to be superior to standard sleeve gastrectomy for weight loss and glucose remission^4^. However, long-term and comparison studies remain unknown and need more research.

Mahdy et al. introduced a technical adjustment to the SG-TB procedure called the single anastomosis sleeve ileal (SASI) bypass^5^. However, there is currently a lack of research on the safety and effectiveness of this procedure. Bile reflux is a commonly recognized issue in the context of SASI^6^. Mohamed et al.^7^ confirmed the presence of biliary reflux and its potential complications in humans following SASI.

It has been suggested that Braun anastomosis may reduce biliary acid reflux in gastrointestinal anastomosis^8^. Based on this, we hypothesized that the addition a Braun anastomosis to the SASI bypass (BTB) may be beneficial in mitigating bile reflux. A recent clinical study^9^, compared BTB to the traditional sleeve gastrectomy with transit bipartition (RYTB) procedure, finding shorter operation time and similar weight loss for the BTB. Further research is needed to confirm the efficacy and safety of BTB.

This study used an obese rat model to compare the RYTB, SASI, and BTB procedures for their effects on weight loss, glucose remission, safety, and most importantly, the biliary reflux to the esophagus for 12 weeks. The potential for biliary reflux to cause carcinogenesis has been established^10^. Esojejunostomy (EJ) in rats could confirm the carcinogenesis potential of biliary reflux on the esophageal mucosa^10,11^. The EJ group is used as a positive control group. We aimed to determine whether Braun anastomosis is necessary for this context, using animal studies as a foundation for future clinical trials due to their faster results compared to clinical trials.

## Results

### Mortality and morbidity

No mortality occurred in the SHAM and EJ groups. The mortality rate in the RYTB and SASI groups was 12.5%, and 25%, i.e., two cases of bowel obstruction in the SASI group and one case of peritonitis (anastomotic fistula) in the RYTB group. We added two rats to the SASI group and one rat to the RYTB group. No mortality occurred afterward. No rats were euthanatized postoperatively.

### Weight loss, food intake, and fasting blood glucose

**Figure 1:**
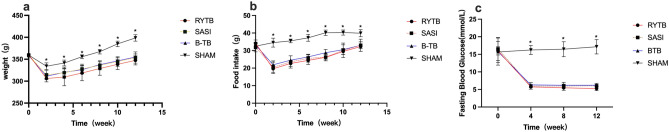
Changes in (**a)** body weight, (**b)** food intake, and (**c)** fasting blood glucose level in all groups. All data are presented as mean ± SD. *Significant RYTB, SASI, and BTB compared with SHAM (p-value < 0.05). *RYTB* sleeve gastrectomy with transit bipartition, *SASI* single anastomosis sleeve ileal bypass, *BTB* single anastomosis sleeve ileal bypass with Braun anastomosis.

For weight changes and food intake, no significant differences were observed in all groups before surgery (Fig. 1a, b). Body weight was significantly lower in the RYTB, SASI, and BTB groups than in the SHAM group postoperatively (p < 0.05). No statistically significant difference in the total weight loss percentage was observed between the RYTB, BTB, and SASI groups postoperatively. Food intake was significantly lower in the RYTB, SASI, and BTB groups than in the SHAM group (p < 0.05) postoperatively, but no significant difference was observed among the three groups.

For the FBG levels, no significant difference was observed in all groups preoperatively (Fig. 1c). The RYTB, BTB, and SASI groups showed significantly lower FBG levels than the SHAM group postoperatively (p < 0.05). The RYTB group showed significantly lower FBG levels than the BTB and SASI groups (p < 0.05). However, no significant differences were observed between the BTB and SASI groups.

### OGTT and ITT

**Figure 2:**
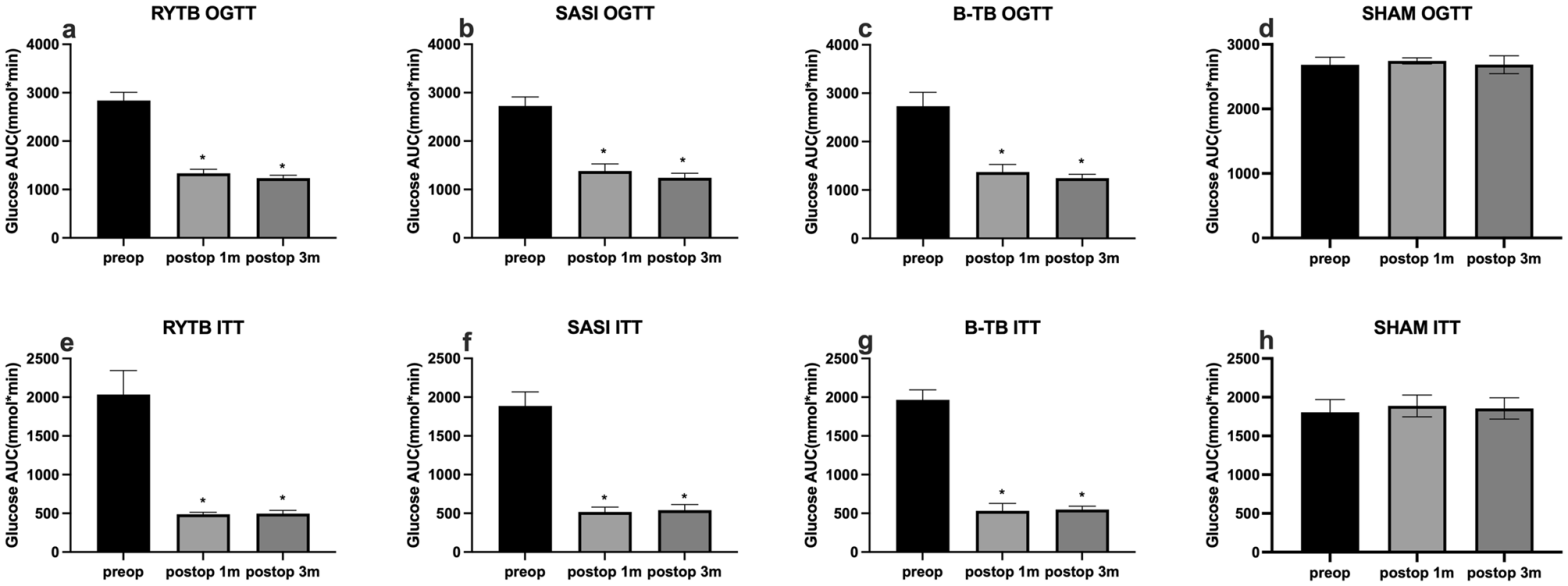
Preoperative (Preop) and postoperative (Postop) oral glucose tolerance test (OGTT) and insulin tolerance test (ITT). All data are presented as mean ± SD. (**a,e)** RYTB OGTT and ITT, respectively; (**b,f)** SASI OGTT and ITT, respectively; (**c,g**) BTB OGTT and ITT, respectively; (**d), (h)** SHAM OGTT and ITT. *Significant difference compared with preoperative level (p-value < 0.05). *RYTB* sleeve gastrectomy with transit bipartition, *SASI* single anastomosis sleeve ileal bypass, *BTB* single anastomosis sleeve ileal bypass with Braun anastomosis.

All groups showed significant improvements in glucose remission postoperatively, except the SHAM group (p < 0.05). However, no significant differences were observed among the three surgery groups at 4 and 12 weeks postoperatively (Fig. 2a–d).

For the ITT results, significant progress in insulin response was observed in the RYTB, BTB, and SASI groups compared to preoperative levels (p < 0.05). Similar to the OGTT results, no significant difference was found in the insulin response among the three groups (Fig. 2e–h).

### Hormonal analysis

**Figure 3:**
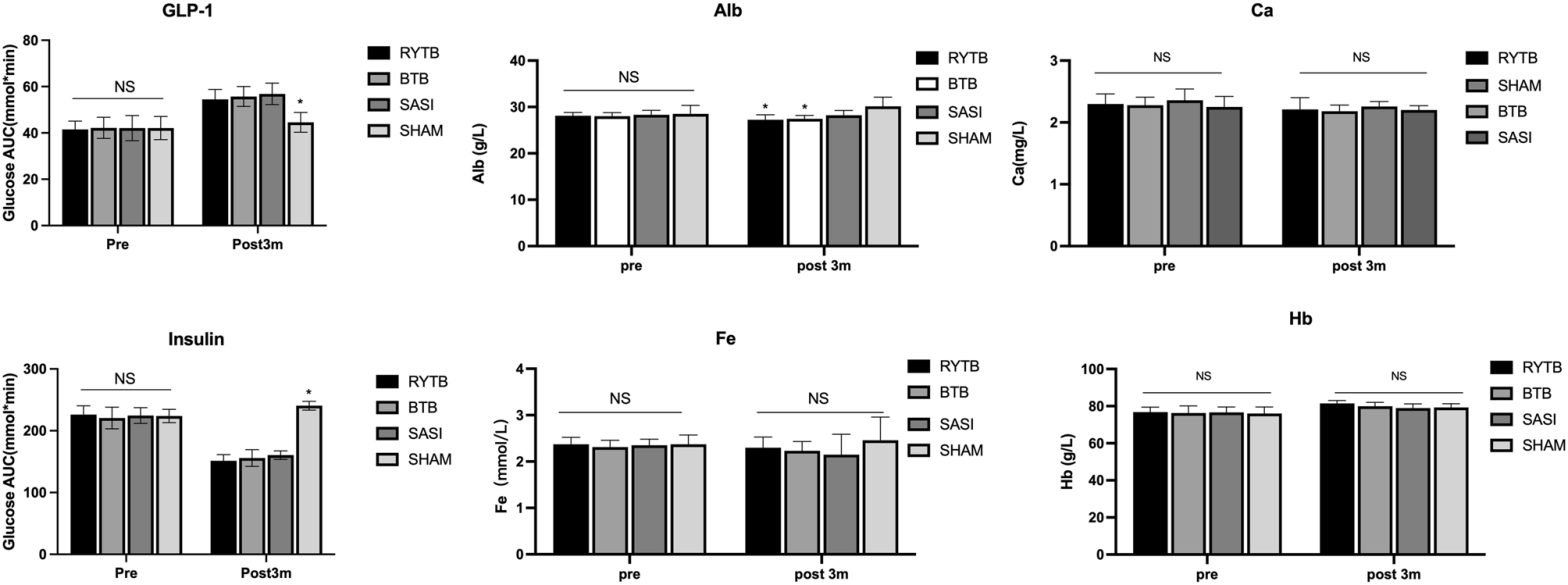
Area under the curve (AUC) of glucagon-like peptide-1 (GLP-1) and insulin levels and nutritional status comparison among groups. (**a)** GLP-1 AUC, (**b)** *Alb* albumin, (**c)** *Ca* calcium, (**d)** insulin AUC,** (e)** *Fe* iron, (**f)** *Hb* hemoglobin. All data are presented as mean ± SD. *Significant compared with other groups postoperatively (p-value < 0.05). *RYTB* sleeve gastrectomy with transit bipartition, *SASI* single anastomosis sleeve ileal bypass, *BTB* single anastomosis sleeve ileal bypass with Braun anastomosis.

No significant differences in GLP-1 and insulin levels were observed preoperatively among the four groups (Fig. 3a, d). The RYTB, BTB, and SASI groups had significantly higher GLP-1 levels postoperatively (p < 0.05). GLP-1 levels were higher in the RYTB group than in the BTB and SASI groups postoperatively, but no statistical difference was observed.

Postoperatively, the RYTB, BTB, and SASI groups had significantly lower insulin levels than preoperative values (p < 0.05). No significant difference was observed in the insulin levels between the surgery groups postoperatively.

### Nutritional states

Postoperatively, the RYTB and BTB groups showed significantly lower Alb levels than the other groups (p < 0.05) (Fig. 3b, c, e, f). Moreover, there were no significant differences in the Hb, calcium, and iron levels among the groups.

### Histology

**Figure 4:**
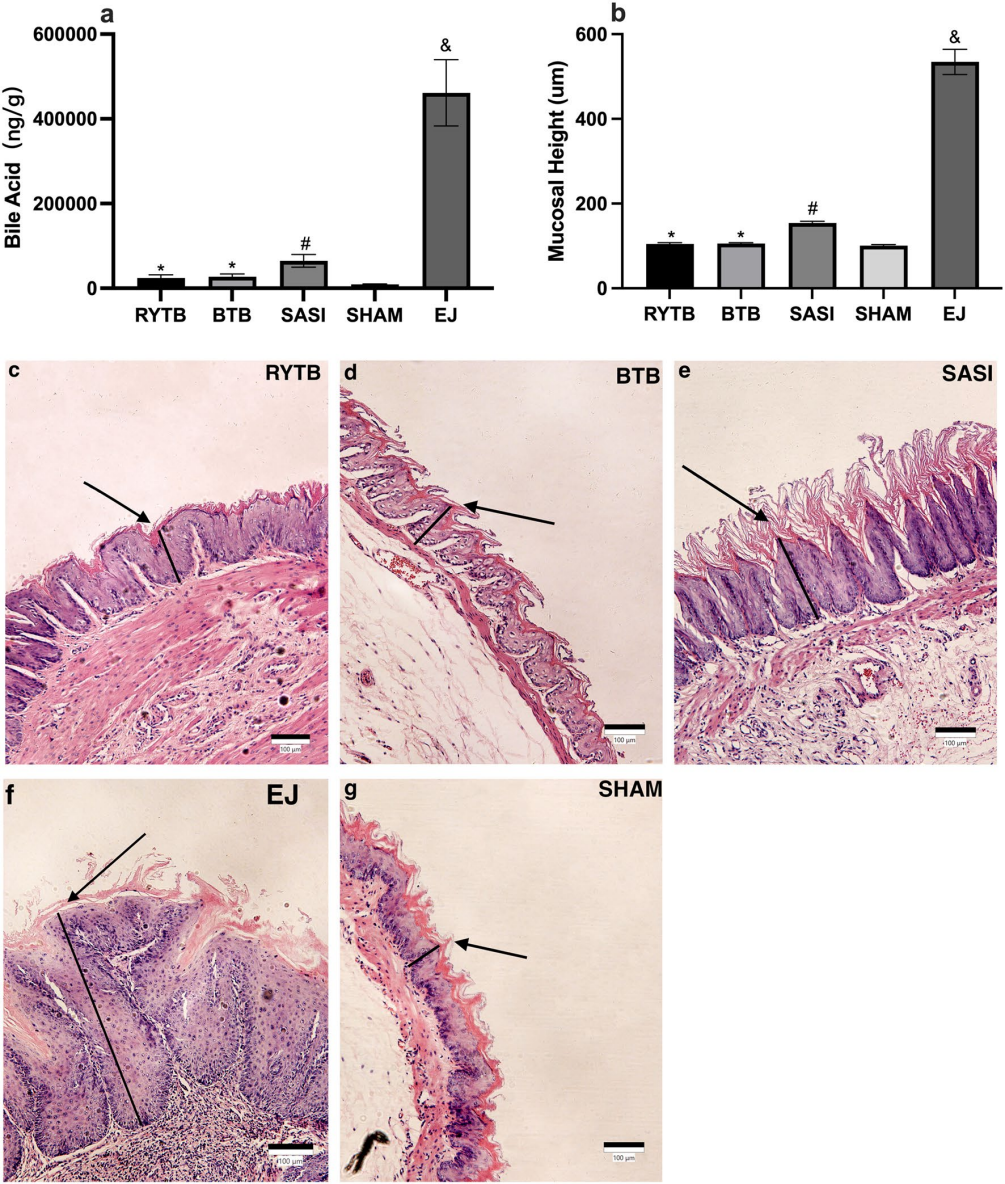
Between-group comparison postoperatively.** (a)** postoperative concentration of mean bile acid in a liquid lavage from esogastric segments if rats had undergone a RYTB, SAIS, BTB, or EJ surgery. Mean bile acid was measured by a HPLC MS/MS system; (**b)** the quantitative mucosal height was observed through the histology. All data are presented as mean ± SD. *Significant RYTB and BTB groups compared with SASI, SHAM, and EJ groups (p-value < 0.05). ^#^Significant SASI group compared with the SHAM and EJ groups (p-value < 0.05). ^&^Significant EJ group compared with other groups (p-value < 0.05). Hematoxylin and eosin (H&E) histology (×100) in (**c)** RYTB, (**d)** BTB, (**e)** SASI, (**f)** EJ, (**g)** SHAM. The black line shows the mucosal height being measured. *RYTB* sleeve gastrectomy with transit bipartition, *SASI* single anastomosis sleeve ileal bypass, *BTB* single anastomosis sleeve ileal bypass with Braun anastomosis.

Postoperatively, EHP was observed in 100% (8/8), 37.5% (3/8), and 12.5% (1/8) of rats in the EJ, SASI, and RYTB groups (Fig. 4c–g). It was not observed in the SHAM or BTB groups. Moreover, we did not observe any intestinal metaplasia, dysplasia, or esophageal cancer postoperatively. The mean mucosal heights were 114.83 ± 3.08 µm, 110.67 ± 1.97 µm, 154.29 ± 4.06 µm, 100.55 ± 2.82 µm, and 534.7 ± 29.76 µm in the RYTB, BTB, SASI, SHAM, and EJ groups, respectively (Fig. 4b). The SHAM group had a significantly lower mucosal height than the other groups (p < 0.05). Mucosal height in the RYTB and BTB groups was significantly lower than that in the SASI group (p < 0.05). However, no significant difference was observed between the RYTB and BTB groups. The EJ group had a significantly higher mucosal height than the other groups.

### Bile acid concentration

Postoperatively, the SHAM group showed a significantly lower mean total bile acid concentration than the other surgery groups (p < 0.05) (Fig. 4a). The bile acid concentration of the RYTB and BTB groups in the esogastric section was 2.5 times higher than that of the SHAM group (24,060 ng/g vs. 9580 ng/g; p < 0.05). The BTB group had 2.7 times more than the SHAM group (27,089 ng/g vs. 9580 ng/g; p < 0.05); the SASI group had 2.7 times more than the RYTB group (65,983 ng/g vs. 24,060 ng/g; p < 0.05) and 2.4 times more than the BTB group (65,983 ng/g vs. 27,089 ng/g; p < 0.05). The EJ group had a significantly higher total bile acid concentration than the other groups (p < 0.05).

## Discussion

Our study compared the bariatric and metabolic effects and safety of three different anastomotic methods of transit bipartition: RYTB, BTB, and SASI procedures. Adding Braun anastomosis to the SASI resulted in less bile reflux and fewer histological changes. Furthermore, we did not observe any significant differences in histological changes and total bile acid concentrations between the RYTB and BTB groups. As for the antidiabetic results, all three surgical procedures achieved excellent diabetes remission but with no significant differences among them. Our study strongly supports Braun anastomosis in patients with severe bile reflux symptoms, but further research is needed.

Obesity has been a worldwide public problem nowadays. Metabolic and bariatric surgery has been acknowledged as the most effective methods for morbid obesity. Sleeve gastrectomy and Roux-en-Y gastric bypass are the most performed procedures in the world^12,13^. After years of modification, single-anastomosis procedures have gained widespread popularity due to their impressive weight loss and metabolic effects^6,14^. However, the use of a single gastrointestinal anastomosis has raised several concerns. In one study, 17 (5.3%) patients experienced biliary reflux due to recurrent bilious vomiting^7^, and Mahdy et al. reported a rate of 5.8%^14^. Chronic bile reflux has been linked to the development of Barrett’s esophagus (BE) and esophageal carcinoma^15^. Bile salts can also stimulate the secretion of pro-inflammatory cytokines, leading to basal cell proliferation and epithelial injury, which can ultimately result in BE and adenocarcinoma. Although the incidence of gastroesophageal carcinoma following SASI is unknown, the potential for gastroesophageal reflux disease (GERD) or esophagitis should be considered. This study is the first to evaluate and compare the outcomes of RYTB, BTB, and SASI operations with a 12-week follow-up period.

To investigate the severity of the bile reflux, we directly measured the mean total bile acid concentration in the esogastric segments. We results showed that bile acid concentrations were significantly lower in the RYTB and BTB groups compared to the SASI group, indicating that the BTB procedure had a significant anti-reflux effect and may provide a protective effect against GERD compared to the initial SASI procedure. The jejunum-jejunum anastomosis may reduce the likelihood of bile acid reflux into the gastric tube and esophagus by allowing bile acid to enter the distal jejunum directly. In one study, Braun anastomosis proved to be valid management for bile reflux after the one anastomosis gastric bypass (OAGB) procedure, with a high success rate of approximately 85%^16^.

The average weight and fast blood glucose in the three surgery groups were statistically lower than the sham group postoperatively. But no statistical differences were observed in weight loss effect and glucose control among the three groups. We also compared the expression of GLP-1 levels in each group. GLP-1, which regulates insulin release, is secreted by L-type endocrine cells in the terminal jejunum, ileum, and colon, and GLP-1 secretion acts on pancreatic β-cells to stimulate the release of insulin, resulting in blood glucose reduction. Numerous studies have identified the promotion of increased GLP-1 secretion as an important mechanism for glucose reduction in bariatric metabolic surgery^17,18^. Although the three different anastomotic methods, they preserve the same length of small intestine, which is 50% of the small bowel length, and the stimulation of GLP-1 is similar. The findings are in line with our expectation.

All surgical procedures presented various insufficiencies in reducing malnutrition. The risk of hypoalbuminemia was higher in the RYTB and BTB groups. The reason considered was that all three surgical groups partially bypass the proximal small intestine, while some food entered the intestine from the normal duodenum. The RYTB and BTB groups had an additional 5 cm of bypassed distal intestine, compared to the SASI group, reducing the absorptive area of the small intestine, which increases the risk of malnutrition. However further clinical trials are required to confirm this conclusion.

Our study had several limitations, including a small sample size, lack of an SG group as a control, short follow-up time, and absence of cancer induction in the EJ group. Future studies should consider these issues and use additional experimental tests to address controversies.

There were no significant differences in weight loss and glucose control between the RYTB, BTB, and SASI groups. However, BTB may be a superior primary procedure as it demonstrated parallel bariatric and metabolic results to the RYTB procedure and a better anti-reflux effect than the SASI procedure. In addition, it is a simpler operation with a lower risk of severe malnutrition, particularly for patients with preoperative GERD, and can be redone after laparoscopic sleeve gastrectomy (LSG) in cases of weight regain and GERD with esophagitis. Further clinical studies are needed to confirm these findings.

## Materials and methods

### Animals

This study was approved by the Ethics Committee of the Research Animal Center of our institution. All applicable institutional and national guidelines for the care and use of animals in our country were followed.

Male Sprague–Dawley rats (ages 8–10 weeks) obtained from our institution’s Research Animal Center were housed in cages with water and food under standard laboratory conditions at a temperature of 21–24 °C. The rats were allowed to acclimate for 1 week before any experimental procedures were performed, and T2DM was induced as previously described^19,20^. High-fat and low-dose streptozotocin were administered. The obese rodent model was checked when the random blood glucose level of the rats was > 16.7 mmol/L for the three following days.

### Surgical procedure

**Figure 5:**
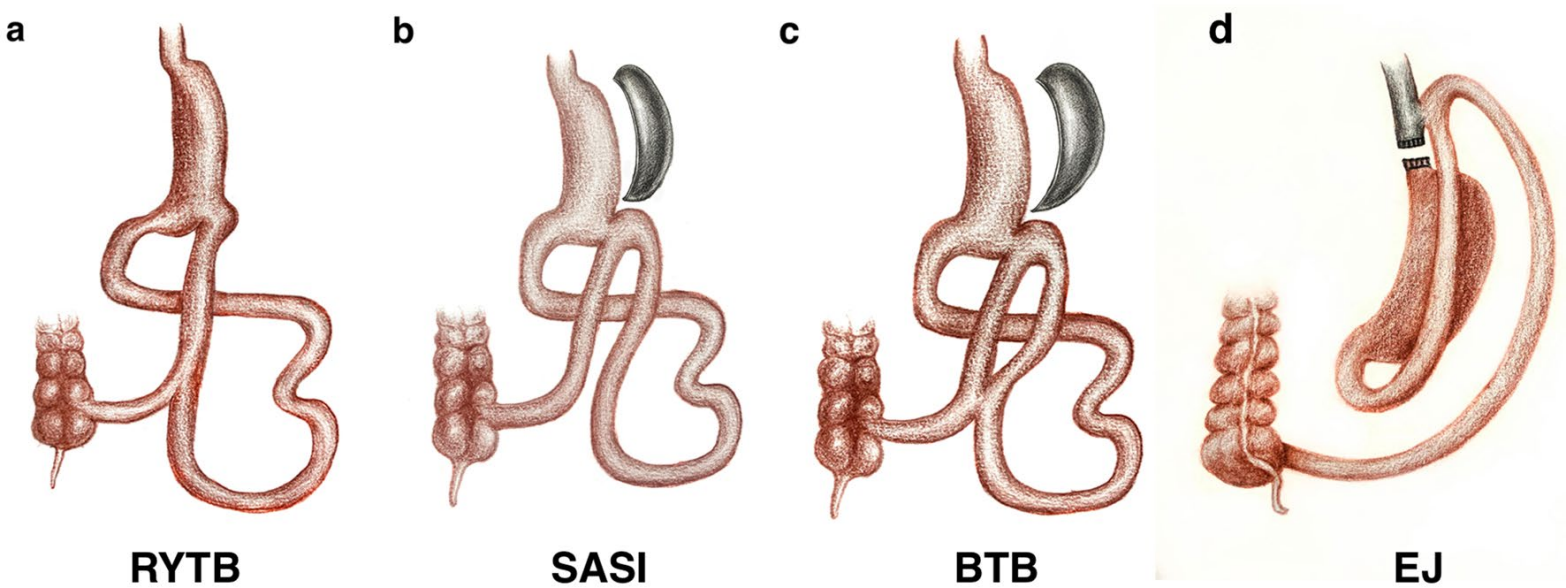
Graphical demonstration of the four surgical models. (**a)** Sleeve gastrectomy with transit bipartition (RYTB), (**b)** single anastomosis sleeve ileal bypass (SASI), (**c**) single anastomosis sleeve ileal bypass with Braun anastomosis (BTB), and (**d**) esojejunostomy (EJ).

Forty-eight rats with diabetes were assigned to the RYTB (n = 12), SASI (n = 12), B-TB (n = 12), SHAM (n = 8), and esojejunostomy (EJ) (n = 12) groups. Fifty-two rats underwent the surgeries (Fig. 5). The mean operation times for the RYTB, BTB, SASI, EJ, and SHAM groups were 55 ± 4, 50 ± 5, 40 ± 5, 37 ± 4, and 7 ± 2 min, respectively. Eight surviving rats from each group were selected after surgery and included in the study.

In the RYTB procedure (Fig. 5a)^21^, a 20–30% gastric pouch was formed. The small intestine was transected at 40 cm from the ileocecal junction. The distal segment was anastomosed to the small gastric pouch, and the proximal segment was anastomosed 5 cm distal to the anastomosis. The length of all anastomoses was approximately 0.3 cm, sutured with 5–0 silk sutures.

For the SASI procedure (Fig. 5b)^22^, after the gastric pouch was created, the small intestine was located approximately 40 cm from the ileocecal junction and anastomosed to the gastric antrum. For the SHAM procedure, similar incisions were made in the stomach and small intestine. The incision was then sutured.

Regarding the BTB procedure (Fig. 5c)^23^, after the SASI procedures were completed, a Braun anastomosis was made at the 5 cm proximal and distal sides of the gastrointestinal anastomosis.

For the EJ procedure (Fig. 5d)^23^, the esophagus was cut to the lowest possible value. The small intestine was located at the ileocecal junction and measured 40 cm at length. A 0.3 cm jejunostomy was created and anastomosed to the esophagus.

The study lasted 12 weeks. Weight changes and food intake were evaluated every 2 days postoperatively. Fasting blood glucose (FBG) levels were evaluated after 12 h of fasting every 2 weeks postoperatively. Oral glucose tolerance test (OGTT, 50% glucose solution, 3 mg/kg) and insulin tolerance test (ITT, insulin, 0.5 IU/kg) were performed preoperatively and at 4 and 12 weeks postoperatively^20^.

Retroorbital blood sampling was performed preoperatively and at 12 weeks postoperatively to evaluate glucagon-like peptide-1 (GLP-1) and insulin levels. In addition, nutritional markers such as hemoglobin (Hb), albumin (Alb), calcium (Ca), and iron (Fe) were measured. Enzyme-linked immunosorbent assay kits were used to evaluate the levels.

### Histology and bile acid measurement

After 12 h of fasting, the rats were sacrificed at 12 weeks postoperatively. Half of the gastroesophageal junctions of the rats were harvested and stored at −80 °C for analysis of bile acids using high-performance liquid chromatography-tandem mass spectrometry^23,24^. The other half of the gastroesophageal junction was formalin-fixed and then routinely embedded in paraffin for further histological analysis. Each slide was stained with hematoxylin and eosin (HES)-Safran. The incidences of esophageal hyperpapillomatosis (EHP), esophagitis, metaplasia, dysplasia, and cancer were investigated. The height of the total esophageal mucosa was measured to determine the influence of bile reflux. EHP and the height of the total esophagus mucosa can laterally reflect the mucosal epithelial damage.

### Statistical analysis

Quantitative values are expressed as mean ± standard error of means (SEMs). GraphPad Prism 8 software was used to evaluate the area under the curve (AUC). One-way ANOVA with repeated measures was used to compare the groups and differences between the means. All values were considered statistically significant at a p-value < 0.05.

### Statement

The study was conducted in strict accordance with ARRIVE guidelines (Animal Research: Reporting of In Vivo Experiments).

## Data Availability

The datasets generated during and/or analyzed during the current study are available from the corresponding author on request.
